# Brain signaling becomes less integrated and more segregated with age

**DOI:** 10.1101/2023.11.17.567376

**Published:** 2023-11-17

**Authors:** Rostam M Razban, Botond B Antal, Ken A Dill, Lilianne R Mujica-Parodi

**Affiliations:** a Laufer Center for Physical and Quantitative Biology, Stony Brook University, Stony Brook, NY, USA; b Dept. of Biomedical Engineering, Stony Brook University, Stony Brook, NY, USA; c Program in Neuroscience, Stony Brook University, Stony Brook, NY, USA; d Dept. of Physics and Astronomy, Stony Brook University, Stony Brook, NY, USA; e Athinoula A. Martinos Center for Biomedical Imaging, Massachusetts General Hospital and Harvard Medical School, Boston, MA, USA; f Dept. of Chemistry, Stony Brook University, Stony Brook, NY, USA

**Keywords:** network neuroscience, integration, segregation, aging, fMRI

## Abstract

The integration-segregation framework is a popular first step to understand brain dynamics because it simplifies brain dynamics into two states based on global vs. local signaling patterns. However, there is no consensus for how to best define what the two states look like. Here, we map integration and segregation to order and disorder states from the Ising model in physics to calculate state probabilities, *P*_int_ and *P*_seg_, from functional MRI data. We find that integration/segregation decreases/increases with age across three databases, and changes are consistent with weakened connection strength among regions rather than topological connectivity based on structural and diffusion MRI data.

## Introduction

Aging is the number one risk factor for almost all neurodegenerative diseases [1]. For every 5 years after the age of 65, the probability of acquiring Alzheimer’s disease doubles [2]. An influential conceptual approach to begin making sense of brain dynamics frames it in terms of a balance between *integrated* and *segregated* network states [3, 4, 5, 6, 7, 8]. On one hand, the brain faces functional pressure to have as many regions directly connected for quick communication. On the other hand, the brain is constrained to minimize metabolic energy consumption because it consumes ten-times more of the body’s energy than expected by mass [9]. Tuning the balance between extensive global signaling, referred to as integration, and limited local signaling, referred to as segregation, optimally compromises between functional and energetic constraints [10, 11, 12, 13]. Although these constraints remain throughout life, aging disrupts their balance.

Previous research found mixed aging results, depending on the metrics used to measure integration and segregation [14, 15, 16, 17]. Although most in the literature use the system segregation metric [14], no consensus exists surrounding integration. In general, the problem facing the integration-segregation framework is that there is no one way to define the two states. Many graph theoretical metrics could potentially be used [18] and it is unclear why one should take precedence over the other, particularly when their aging outcomes are mutually inconsistent. There is a need to more fundamentally define integration and segregation to transform it from a proxy to a physical quantity.

Here, we provide a physical foundation for the framework by applying the mean field Ising model to treat integration and segregation as physical 2-phase systems like magnets and liquids. After demonstrating that the Ising model can capture global brain dynamics as measured by functional MRI once the effective number of nodes is properly set, we proceed to calculate probabilities of being in the integrated or segregated states and find that younger and older brains are bounded by optimal and random signaling, respectively. We then explore diffusion and structural MRI data to ask if the age-related changes in signaling are due to changes in topological network connectivity.

### Applying the Ising model to fMRI

**Figure 1:**
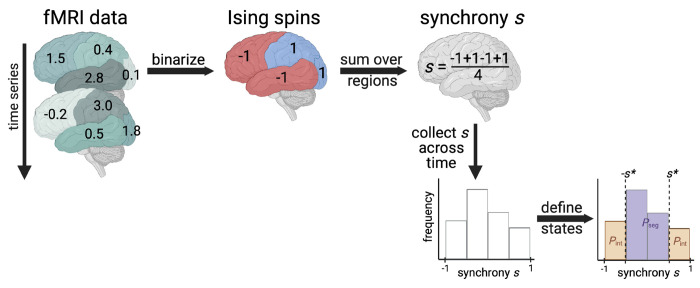
Calculating the probability that the brain exhibits integrated or segregated dynamics (*P*_int_ or *P*_seg_). The schematic demonstrates the procedure for one individual’s fictitious functional MRI scan with 4 brain regions and only two time points shown. First, we binarize data based on nearest neighbor scans in time. If functional MRI (fMRI) signal increases, a value of 1 is assigned; decreases, −1. Then, we calculate the average spin state of the brain, called synchrony. Finally, we collect synchrony values across the entire time series to create a synchrony distribution. We appropriately set the synchrony threshold based on Ising model theory to delineate between integrated and segregated microstates. Additional details can be found in the Methods. Figure created with Biorender.com.

We model human brain signaling patterns obtained from data sets of resting-state functional MRI (fMRI). As in previous work [19], we capture those patterns using the Ising model, a widely used theoretical method for expressing macroscale behaviors in terms of interactions among many underlying microscale agents [20]. We first transform the continuous fMRI data into representation as discrete Ising spins via binarization of the data (Figure 1). That is, we reduce the state of the region as simply being negative or positive, following the way that Ising models have spins of either −1 or 1 (details in Methods). Second, we calculate the *synchrony* by summing over all spins in a given time interval and dividing by the total number of spins (Figure 1). Before proceeding to the last step of calculating the probability that the brain is in the segregated state *P*_seg_,^1^ we first check whether the model so obtained can capture the experimental synchrony distributions.

## Results

### The number of functionally effective brain regions

**Figure 2:**
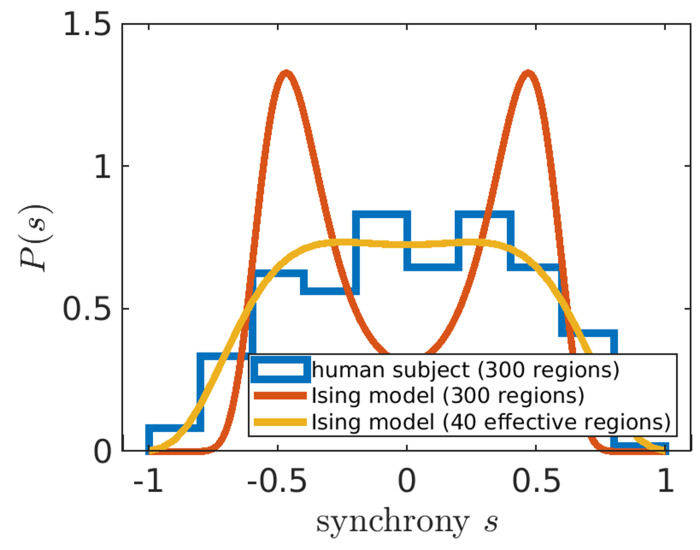
Adjusting the number of brain regions (*N*_eff_) helps capture experiment. The modified Ising model with *N*_eff_ = 40 (yellow line) better captures the synchrony distribution (blue histogram) of an arbitrarily chosen individual in the Cambridge Centre for Ageing data set (subject id: CC110045). The orange line corresponds to the Ising model with *N* equal to the number of regions as in the Seitzman atlas [24], which is used in fMRI preprocessing.

An Ising model only considering pairwise interactions typically has one quantity of interest. The strength of connection λ between any two regions^2^ corresponds to the degree to which signals between any two brain regions are correlated. However, we find that a naive fit of λ based on maximum entropy fails to capture the synchrony distribution from fMRI data (Figure 2, orange). To improve upon a standard Ising model approach, here we introduce a hyper-parameter *N*_eff_. Brain atlas parcellations provide *N* brain regions, however, those *N* regions must be identically distributed across time for the Ising model to apply. We find that when setting *N* to a lower value *N*_eff_, fixed for all individuals within a data set, the Ising model accurately captures synchrony distributions (Figure 2). The optimal value of *N*_eff_ = 40 is determined by scanning across *N*_eff_ multiples of 5 to find which best captures the next order moment not fit by our maximum entropy setup across all individuals (Methods, Figure 6). For our particular preprocessing (Methods), we find that *N*_eff_ = 40 for individuals in the Cambridge Center for Ageing (CamCAN) [21] and the Human Connectome Project Aging (HCP) [22]. For the UK Biobank (UKB) [23], *N*_eff_ = 30 performs best (Figure 6).

Based on identified *N*_eff_ hyper-parameter values, brains act as if they have a few tens of functional units. If different preprocessing decisions are considered, such as atlas resolution, *N*_eff_ values are still within an order of magnitude. At the voxel-level (*N* = 125, 879), we obtain an *N*_eff_ value of 65 for CamCAN and 125 for HCP using the same procedure as for the Seitzman atlas (*N* = 300) considered in the previous paragraph (Figure S2). Future work will pinpoint how *N*_eff_ depends on preprocessing to enable a future study creating a physics-based parcellation of the brain.

### The aging brain becomes functionally more segregated

*P*_seg_ is the relative number of time points for which the absolute value of synchrony is less than the delineating threshold between segregated and integrated microstates (Figure 1). The synchrony threshold s* is set such that at the Ising model’s critical point in connection strength λ, *P*_seg_ equals 1/2 for the ideal synchrony distribution based on Ising model theory (Methods). For CamCAN and HCP, s* = 0.33 because *N*_eff_ = 40 for both data sets. For UKB, s* = 0.36 (Table S1).

**Figure 3:**
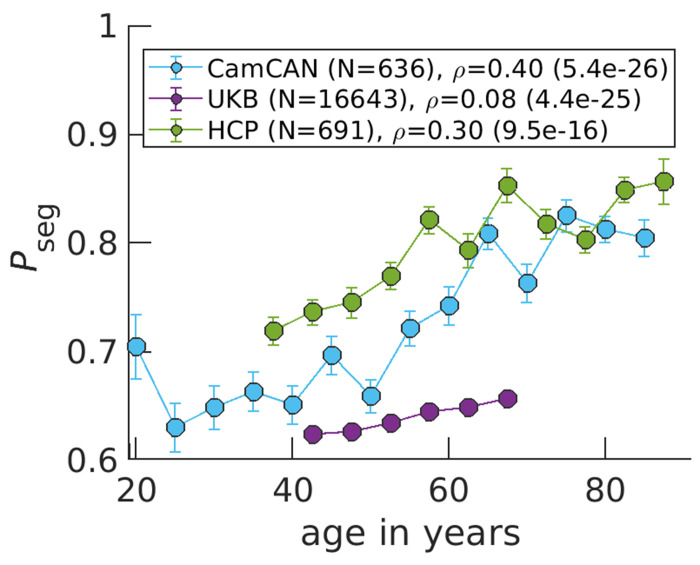
*P*_seg_ rises in aging brains across three data sets. Data points correspond to medians, while error bars correspond to standard errors for bins of 5 years. The variable *ρ* corresponds to the Spearman correlation coefficient between age and *P*_seg_ calculated over all N individuals, with the p-value in parenthesis.

Across the three publicly available data sets, we find that the balance shifts towards more segregation at older ages (Figure 3). Note that if we plotted *P*_int_ rather than *P*_seg_, Figure 3 would be horizontally flipped, where *P*_int_ goes from high to low values as a function of increasing age because *P*_seg_ + *P*_int_ = 1. There is large variation among subjects (Figure S3). However, the correlation between age and *P*_seg_ is significant with the largest coefficient being 0.40 for CamCAN, while the lowest being 0.08 for UKB. Discrepancies in study designs may explain correlation magnitude differences: CamCAN and HCP are designed to study healthy aging [25, 26], while the goal of UKB is to identify early biomarkers for brain diseases [27].

Informed by the Ising model, increases in segregation result from network reorganization to more local signaling because of weakened connection strength between regions. Interestingly, younger individuals exhibit segregation behavior closer to the Ising model’s critical point of connection strength (Figure S4). At the critical point, we define *P*_seg_ = 1/2 (Methods) and find experimental *P*_seg_ values closer to 1/2 for younger individuals (Figure 3). Older individuals, on the other hand, approach *P*_seg_ = 1 on average. This limit corresponds to functionally uncoupled brain regions that are randomly activating. Our results support the critical brain hypothesis that healthy brains operate near a critical point [28, 29, 30, 31] and implicate aging as pushing brain dynamics further away from criticality.

### Changes in brain dynamics are not due to structural degradation

**Figure 4:**
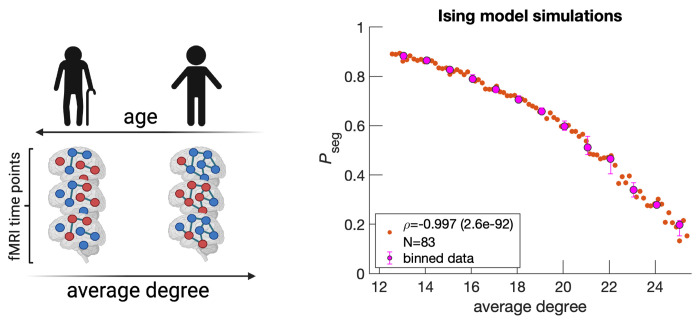
Simulating the random removal of edges results in *P*_seg_ increases. Five edges are randomly removed from a starting diffusion MRI structure (arbitrarily chosen UK Biobank individual, subject ID: 6025360). An Ising system is simulated and spin states, denoted by dark blue and red node colors, are recorded across 2500 time steps to calculate *P*_seg_. Then, the entire procedure is repeated for the updated structure, for a total of 83 times (Methods). Orange data points on the right plot correspond to individual Ising systems, where N reflects the total number. The variable *ρ* corresponds to the Spearman correlation coefficient calculated over all orange data points between average degree and *P*_seg_, with the p-value in parenthesis. Magenta data points correspond to medians, while error bars correspond to upper and lower quartiles for bin sizes of one degree. The schematic on the left is created with Biorender.com.

In the previous subsection, we discussed the disruption of the integration and segregation balance from the perspective of phase transitions in physics. Here, we explore the physiological mechanism underlying increasing segregation in the aging brain. We consecutively simulate the Ising model on a hypothetically degrading brain structure and show that random removal of edges yields qualitatively similar results to those of fMRI (Figure 4). Note that Figure 4 is horizontally flipped from those of *P*_seg_ (Figure 3) because average degree is on the x-axis. It is presumed that edges are lost as age increases. In Figure 4, edges are lost linearly in time, however, more complicated monotonic functions can be employed to yield a quantitative match with experimental data in Figure 3. We can also capture variability among individuals by assuming connection strengths within an individual are drawn from a distribution, rather than all being equal (Figure S6).

We now begin to investigate possible mechanisms of connection degradation. First, we find that our simulation is agnostic to the detailed mechanism of connection degeneration because connection strength is essentially modulated by the probability that a given edge exists (Figure S7). In other words, the simulation cannot inform whether connections are degraded based on some targeted property. Thus, we turn to MRI and diffusion MRI data from UKB to investigate possible properties being degraded with age. In Figure S8, we confirm that white matter volume decreases as a function of adult age, as previously reported [32, 33, 34]. However, this decrease does not correspond to a loss of anatomical connections because we find that neither average degree, average tract length nor average tract density monotonically decrease with age when analyzing diffusion MRI scans using the Q-Ball method (Figure S9). This seems to contradict previous findings which report decreases [35, 36]. However, previous results employed the more simple diffusion tension imaging (DTI) method which is known to be less accurate at performing tractography [37, 38, 39]. When rerunning our analysis for DTI, we can reproduce previously reported tract properties’ anticorrelations with age (Figure S9).

We propose that the observed reduction in white matter volume (Figure S8) and brain dynamics changes corresponds to less myelin covering axons as function of age. Despite conclusively rejecting anatomical connections as a possible mechanism in the previous paragraph, it remains inconclusive whether myelin underlies trends because we are not aware of such data being publicly available. Although axons are still physically present, myelin coverage disruption causes regions to no longer be functionally connected because signals do not arrive on time. Previously reported results from Myelin Water Imaging confirm reduction in myelin at advanced ages [40, 41]. We also investigated whether degraded functional connections are likely to be longer than average with age, as previously reported for certain brain regions [42]. Although we indeed find that the average correlation of the 25% longest connections is slightly more strongly anticorrelated with age compared to the average correlation of the 25% shortest connections for CamCAN (Figure S10, left), we find the opposite trend for HCP (Figure S10, right). Thus, myelin reduction does not seem to have a stronger impact on longer connections and conclude that the loss of functional connections happens randomly with respect to length at the brain-wide scale.

## Discussion

We apply the mean field Ising model to physically quantify the integration and segregation framework at the emergent scale of the whole brain. From resting-state fMRI scans across three publicly available data sets, we find that brain dynamics steadily becomes more segregated with age. Physically, aging leads to brain dynamics moving further away from its optimal balance at the critical point. Physiologically, analyses of white matter properties point to random functional connection losses due to myelin degeneration as the possible culprit for more segregated dynamics.

The Ising model and integration-segregation frameworks are considered as the simplest approaches to capture dynamics in their respective fields. Thus, it is fitting to map segregated and integrated states in neuroscience to disordered and ordered Ising model phases in physics, respectively. One general challenge in applying graph theory to MRI-level data is identifying what constitutes a node [8, 43, 44, 45, 46, 47]. We identify the best number of effective brain regions *N*_eff_ such that the Ising model accurately captures individuals’ synchrony distributions across the corresponding data set. Future work will utilize *N*_eff_ calculations to guide the creation of a parcellation in which brain regions are constrained to be physically independent based on their collective functional activity.

The field is inundated with integration and segregation metrics that have different aging trends. Our metric is mechanistically based on the connection strength between regions and stands out for two reasons. First, *P*_seg_ and *P*_int_ are naturally at the emergent scale of the brain. We do not calculate a local property and then average over nodes to yield a brain-wide value ([13] also has this strength). Second, *P*_seg_ and *P*_int_ are directly related because *P*_seg_ + *P*_int_ = 1. Most integration and segregation metrics [3, 18, 14, 13] are not defined to be anti-correlated^3^.

Taken together, it is not surprising that *P*_seg_ and *P*_int_ results are not consistent with some previous reports. For example, a property called system segregation, defined as the difference between inter- and intra-correlations among modules, was found to decrease with age [14]. Although most report that segregation decreases with age, regardless of the specific metric [14, 48, 49, 17] (see [16] for an exception), integration trends are less clear. Global efficiency, taken from graph theory, was found to increase with age [14, 50]; however, others found different integration metrics decreasing with age [51, 52, 17], consistent with results reported here.

The utility of the integration-segregation framework lies in its simplicity. However, its simplicity has led to various heuristic definitions that have qualitatively different aging trends. By physically defining integration and segregation based on connection strength between regions, we provide an interpretable foundation for more detailed studies going beyond the two-state approximation to investigate brain dynamics.

## Methods

### fMRI preprocessing

We access three publicly available resting-state functional MRI data sets: Cambridge Centre for Ageing (CamCAN) [21], UK Biobank (UKB) [23], and Human Connectome Project (HCP) [22]. Acquisition details such as field strength and repetition time can be found in Table S2.

UKB and HCP fMRI data are accessed in preprocessed form (for details see [23] and [53, 54], respectively). We preprocessed CamCAN data as done in our previous work [19]. For all three data sets, the cleaned, voxel space time series are band-pass filtered to only include neuronal frequencies (0.01 to 0.1 Hz) and smoothed at a full width at half maximum of 5 mm. Finally, we parcellate into 300 regions of interest according to the Seitzman atlas [24]. For our voxel-wide analysis presented in the Supporting Information, we do not parcellate and just consider gray mater voxels by masking.

Applying the Ising model requires data to only take two possible values: −1 or 1. After performing the preprocessing outlined in the previous paragraph, we binarize the continuous signal for a given region based on the sign of the slope of subsequent time points [19]. We previously showed that such binarization still yields similar time correlations as that of the originally continuous data [19].

Finally, we only consider brain scans that have the same number of measurements as the predominant number of individuals in the respective data set (Table S2). If the fitted connection strength parameter λ is less than 0, reflecting a nonphysical value, we do not include the individual’s brain scan in our analysis. In the HCP dataset, we excluded individuals aged 90 years or older from our analyses because their exact age, considered protected health information, was not available.

### Identifying the *N*_eff_ hyper-parameter

**Figure 5:**
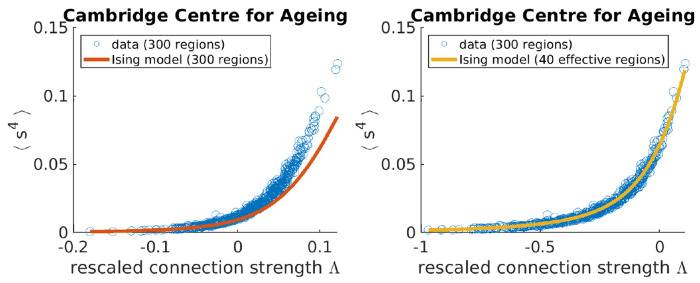
Adjusting the effective number of brain regions (*N*_eff_) helps capture synchrony distributions’ variances across individuals in the Cambridge Centre for Ageing data set. Each data point corresponds to an individual.

In Figure 2, our maximum entropy fit (orange line) fails to qualitatively capture the synchrony distribution for an arbitrary individual. To see if we can rescue the maximum entropy fit, we replace *N* with *N*_eff_ (Equation S1). In the right plot of Figure 5, we demonstrate that a fully connected Ising model with *N*_eff_ = 40 accurately captures the fourth moment of synchrony 〈s^4^〉 across all individuals in CamCAN, preprocessed under the Seitzman atlas. Note that *N*_eff_ is not a parameter like Λ; rather it is a hyper-parameter because it takes the same value across all individuals within the data set. *N*_eff_ is necessary because the Ising model systematically underestimates 〈s^4^〉 when Λ > 0 (left plot of Figure 5). Note that Λ corresponds to rescaling λ such that Λ = 0 is at the critical point (Equation S13).

**Figure 6:**
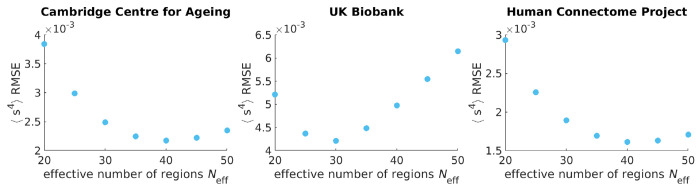
The effective number of regions *N*_eff_ is identified by minimizing the root mean square error (RMSE) of the fourth moment of synchrony between theory and experiment across all individuals. Each data point corresponds to the sum over all individuals’ RMSEs in the respective data set.

To identify *N*_eff_ = 40 as the best value, we perform a parameter scan over multiples of 5 and identify *N*_eff_ at which the root mean square error (RMSE) between 〈*s*^4^〉_exp_ and 〈*s*^4^〉_model_ is minimized (Figure 6). We choose the fourth moment because it is the next order moment that our maximum entropy fit does not constrain^4^. We could also use other metrics to identify *N*_eff_, such as the Kullback-Leibler divergence.

### Calculating *P*_seg_

The probability of the brain network being in the segregated state is the sum over all microstates corresponding to the segregated state.


(1) $$P_{\text{seg}}=\frac{1}{Z}\overset{N_{\text{eff}}{\text{s}}^{*}}{\underset{n=-N_{\text{eff}}{\text{s}}^{*}}{\sum }}P(n)$$

(2) $$P_{\text{seg}}=\frac{1}{Z}\overset{N_{\text{eff}}{\text{s}}^{*}}{\underset{n=-N_{\text{eff}}{\text{s}}^{*}}{\sum }}\left(\begin{array}{c} N_{\text{eff}}\\ \left(N_{\text{eff}}+n\right)/2 \end{array}\right)e^{\lambda n^{2}/N_{\text{eff}}^{2}}$$

In the second line, the expression for *P*(*n*) is the solution to the mean field Ising model (Equation S3). The constant s* is the synchrony threshold for which segregated and integrated microstates are delineated. We set s* such that *P*_seg_ = 1/2 when Λ = 0 according to theory. More specifically, we numerically calculate *P*_seg_(Λ = 0) for a given *N*_eff_ and extrapolate to find s* (Figure S5). Proper calibration ensures that the theory is accurate and enables apples to apples *P*_seg_ comparisons across data sets with different *N*_eff_. The list of s* values for the three publicly available data sets studied can be found in Table S1.

### Ising model simulation

We simulate the Ising model on an initial structure informed by diffusion MRI under the Harvard-Oxford atlas [55] (64 regions) for an arbitrarily chosen UK Biobank individual (subject ID: 6025360). Edge removal procedures all begin with this starting structure. If no edge exists between two regions, then the regions are uncoupled. If an edge does exist, then regions *i* and *j* are coupled and contribute λ * *σ_i_* * *σ_j_* to the system’s energy; where λ corresponds to the connection strength and *σ* corresponds to the spin state of the corresponding region (−1 or 1). λ is set to 0.0084, which is above its critical point (starting *P*_seg_ ≈ 0.2).

The simulation for a given structure starts by randomly assigning the 64 nodes up or down spins. Then, for each time step, we attempt 10 spin flips 64 times for a total of 2500 time steps. Spin flips are accepted according to the Metropolis-Hastings algorithm [56]. The exact number of spin flip attempts or total time points does not matter, as long as equilibrium is reached. For example, we find that for λ values larger than those presented in the text, synchrony distributions become asymmetric and exhibit only one of the two peaks corresponding to the integrated state because of the high kinetic barrier of going from all down spins to all up spins.

Although the starting structure is informed by diffusion MRI, resulting structures after edge removals are based on the posited removal strategy. Edges informed by dMRI are undirected and removal maintains undirectedness. Effectively two times as many edges are removed because both forward and backward edges are concurrently eliminated.

### Diffusion MRI analysis

Diffusion MRI processing to obtain structural information like tract length and streamline count, which we call tract density, are outlined in our previous work [57]. Briefly, we take preprocessed dMRI scans from the UK Biobank [27] and calculate connectivity matrices using the Diffusion Imaging in Python software [39]. We input the Talairach atlas [58] to distinguish between white and gray matter. We perform deterministic tractography and reconstruct the orientation distribution function using Constant Solid Angle (Q-Ball) with a spherical harmonic order of 6 [59]. For Figure S9, we also do reconstruction using diffusion tensor imaging [39]. To generate the starting structure for Ising model simulations, we input the Harvard-Oxford atlas for tractography because it parcellates the brain into a fewer number of regions, making it more computationally tractable to carry out simulations.

### Code and data availability

Scripts necessary to reproduce figures and conclusions reached in the text can be found at github.com/rrazban/2state_brain. Please refer to the respective publicly available diffusion MRI data set to access previously published data (CamCAN, UKB and HCP) [21, 22, 23].

## Supplementary Material

1

## Footnotes

1 *P*_int_ trivially relates to *P*_seg_ because *P*_int_ + *P*_seg_ = 1.

2 Under standard Ising model notation, λ = *J/T*, where *J* corresponds to the coupling constant and *T* is the temperature of the bath.

3 This can be advantageous because greater complexity can be captured [8].

4 It is not the third moment because the distribution is assumed to be even as indicated by our prior (Equation S1).
