# Supplementary material for: Brain signaling becomes less integrated and more segregated with age
Source: bioRxiv. 2024 May 2:2023.11.17.567376. Originally published 2023 Nov 17. Preprint. [Version 2] doi: 10.1101/2023.11.17.567376 (PMC10680817; doi:10.1101/2023.11.17.567376)
Supplement: Supplement 1 [file NIHPP2023.11.17.567376v2-supplement-1.pdf]

bioRxiv preprint doi: https://doi.org/10.1101/2023.11.17.567376; this version posted May 2, 2024. The copyright holder for this preprint (which was not certified by peer review) is the author/funder, who has granted bioRxiv a license to display the preprint in perpetuity. It is made available under aCC-BY 4.0 International license.

SUPPORTING INFORMATION

Deriving the Ising model with N_{eff}

Many have derived the probability distribution of the mean field Ising model, otherwise known as the fully connected or Curie-Weiss Ising model (Friedli & Velenik, 2017; Kochmański, Paszkiewicz, & Wolski, 2013; Weistuch et al., 2021). Here, we demonstrate how to introduce N_{eff} in a Maximum Entropy framework. The tricky part is that N_{eff} defines the state space over which the probability distribution is summed.

Adding a global pairwise correlation constraint, we obtain the following Lagrangian function $\mathcal{L}$ over the net displacement of spin states $n = \sum_i^{N_{\text{eff}}} \sigma_i$, where σ_i can take a value of 1 or -1.

$$\mathcal{L} = \sum_n^{N_{\text{eff}}} P(n) \ln \frac{P(n)}{q(n)} + \alpha \left(\sum_n^{N_{\text{eff}}} P(n) - 1 \right) + \lambda \left(\frac{1}{(N_{\text{eff}})^2} \sum_n^{N_{\text{eff}}} n^2 P(n) - \langle s^2 \rangle_{\text{exp}} \right) \tag{S1}$$

P is the probability distribution. q corresponds to the prior and is set to the binomial distribution $\binom{N}{(N+n)/2} 2^{-N}$, where the binomial coefficient captures the number of ways individual spins can organize for a given n. α and λ correspond to the Lagrange multipliers that enforce the constraints that the probability distribution is normalized and the mean pairwise correlation equals $\langle s^2 \rangle$, respectively. The variable s corresponds to the synchrony, or commonly referred to as the magnetization in ferromagnetic applications, and is limited to vary from -1 to 1 because $n = N_{\text{eff}} s$. This is the reason N does not have to be the same for $\langle s^2 \rangle_{\text{exp}}$ and $\langle s^2 \rangle_{\text{model}}$; $\langle s^2 \rangle$ is always bounded between -1 and 1.

Maximizing the Lagrangian function (Equation S1) with respect to P, we obtain the following distribution:

$$P(n) = Z^{-1} \binom{N_{\text{eff}}}{(N_{\text{eff}} + n)/2} e^{\lambda n^2 / N_{\text{eff}}^2} \tag{S2}$$

$$P(s) = Z^{-1} \binom{N_{\text{eff}}}{N_{\text{eff}}(1 + s)/2} e^{\lambda s^2} \tag{S3}$$

Z corresponds to the partition function and ensures that P is normalized. The α Lagrange multiplier is not present in the final expression because it is subsumed by Z.

bioRxiv preprint doi: https://doi.org/10.1101/2023.11.17.567376; this version posted May 2, 2024. The copyright holder for this preprint (which was not certified by peer review) is the author/funder, who has granted bioRxiv a license to display the preprint in perpetuity. It is made available under aCC-BY 4.0 International license.

Ising model phase transitions

The Landau model is a general formulation to study phase transitions (Dill & Bromberg, 2012; Landau, 1937). It takes the following form,

$$F(T) = A(T - T_c)\eta^2 + B\eta^4 \tag{S4}$$

η corresponds to the order parameter. F is the free energy and can be expressed as the probability for being in microstate i by the following relationship $F_i = k_b T \ln P_i$. T is the temperature and T_c corresponds to the critical temperature at which a second-order phase transition occurs. A and B are constants.

Here, we will express the Ising model's probability distribution (Equation S3) in terms of the Landau formalism (Equation S4) by approximating the binomial coefficient as an exponential to order (s^4). For brevity, we will write N to represent N_{eff}. First, we use Stirling's approximation to expand the binomial coefficient.

$$\binom{N}{N(1+s)/2} = N! \left[(N(1+s)/2)! \, (N(1-s)/2)! \right]^{-1} \tag{S5}$$

$$\approx N^N \left[\left(\frac{N(1+s)}{2}\right)^{\frac{N(1+s)}{2}} \left(\frac{N(1-s)}{2}\right)^{\frac{N(1-s)}{2}} \right]^{-1} \tag{S6}$$

$$= N^N \left[\left(\frac{N}{2}\right)^{\frac{N(1+s)}{2}} (1+s)^{\frac{N(1+s)}{2}} \left(\frac{N}{2}\right)^{\frac{N(1-s)}{2}} (1-s)^{\frac{N(1-s)}{2}} \right]^{-1} \tag{S7}$$

$$= N^N \left[\left(\frac{N}{2}\right)^{N} ([1+s][1-s])^{N/2} \left(\frac{1+s}{1-s}\right)^{Ns/2} \right]^{-1} \tag{S8}$$

$$= 2^N \left[\frac{1}{1-s^2} \left(\frac{1-s}{1+s}\right)^{s} \right]^{N/2} \tag{S9}$$

To make further headway, we assume that s approaches 0 and expand Equation S9 to order s^4.

bioRxiv preprint doi: https://doi.org/10.1101/2023.11.17.567376; this version posted May 2, 2024. The copyright holder for this preprint (which was not certified by peer review) is the author/funder, who has granted bioRxiv a license to display the preprint in perpetuity. It is made available under aCC-BY 4.0 International license.

$$\binom{N}{N(1+s)/2} \approx 2^N \left[1 - s^2 - \frac{1}{3}s^4\right]^{N/2} \tag{S10}$$

Next, we assume N is large and express the term under the brackets as an exponential.

$$\binom{N}{N(1+s)/2} \approx 2^N \exp\left[-\frac{N}{2}s^2 - \frac{N}{12}s^4\right] \tag{S11}$$

We can insert our approximate expression for the binomial coefficient back into $P(s)$ (Equation S3) and obtain,

$$P(s) \approx Z^{-1}2^N \exp\left[\left(\lambda - \frac{N}{2}\right)s^2 - \frac{N}{12}s^4\right] \tag{S12}$$

Note that Equation S12 (after transforming into free energy space) maps onto Landau theory (Equation S4). s corresponds to the order parameter and $\lambda_c = N/2$. At $\lambda = \lambda_c$, $P(s)$ switches from unimodal to bimodal, corresponding to a second order phase transition. We report a rescaled version of λ called Λ in Figure 5 and in other places in the Supporting Information to easily gauge how far an individual's connection strength is from the critical point.

$$\Lambda = \frac{\lambda - \lambda_{\text{critical}}}{\lambda_{\text{critical}}} \tag{S13}$$

Alternative N_{eff} fitting approach

Rather than choose one N_{eff} for all individuals in the data set as done in the main text, we could fit N_{eff} for each individual. Figure S1 demonstrates that such a procedure results in N_{eff} values that are highly linearly related with λ. In other words, more precise N_{eff} fits do not provide any more insight than maximum entropy fits of λ for all individuals in a data set under one optimal N_{eff}.

bioRxiv preprint doi: https://doi.org/10.1101/2023.11.17.567376; this version posted May 2, 2024. The copyright holder for this preprint (which was not certified by peer review) is the author/funder, who has granted bioRxiv a license to display the preprint in perpetuity. It is made available under aCC-BY 4.0 International license.

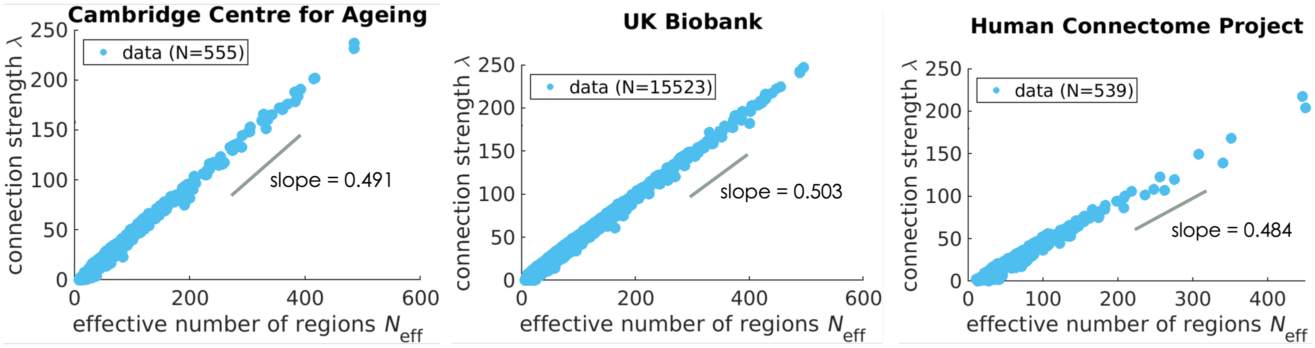

Figure S1. Treating N_{eff} as a parameter and fitting it per individual yields a strong correlation with λ. Each point reflects an individual brain scan and N reflects the total number analyzed. The value of the slope corresponds to that of the best-fit line for the data and is close to the predicted value of 0.5 (Equation S15). N is smaller than that of Figure 3 because some scans failed to have a minimum $\langle s^4 \rangle$ RMSE within the explored bounds of N_{eff} (4-500) or λ values were nonphysical by being less than 0.

The N_{eff}-λ relationship can be reasoned from the analytical expression for $P(s)$ (Equation S12). When $\Lambda < 0$, which many individuals satisfy (Figure S10), $P(s)$ is well-approximated as a Gaussian.

$$P(s) \propto \exp\left[\left(\lambda - \frac{N_{\text{eff}}}{2}\right) s^2\right] \tag{S14}$$

Thus, the analytical form for $\langle s^2 \rangle$ is:

$$\langle s^2 \rangle = -\frac{1}{2\lambda - N_{\text{eff}}} \tag{S15}$$

Since λ is fit in the Maximum Entropy framework to exactly match $\langle s^2 \rangle$, Equation S15 indicates that a larger N_{eff} requires a larger λ for a fixed $\langle s^2 \rangle$. Indeed, we find in Figure S1 that the best fit line of the N_{eff}-λ relationship has an approximate slope of 0.5, in agreement with Equation S15.

More supporting information

bioRxiv preprint doi: https://doi.org/10.1101/2023.11.17.567376; this version posted May 2, 2024. The copyright holder for this preprint (which was not certified by peer review) is the author/funder, who has granted bioRxiv a license to display the preprint in perpetuity. It is made available under aCC-BY 4.0 International license.

Table S1. Data set values for P_{seg} calculations under our particular fMRI preprocessing procedure (Methods).

Data set	effective number of regions N_{eff}	synchrony threshold s^*
Cambridge Centre for Ageing	40	0.334
UK Biobank	30	0.357
Human Connectome Project	40	0.334

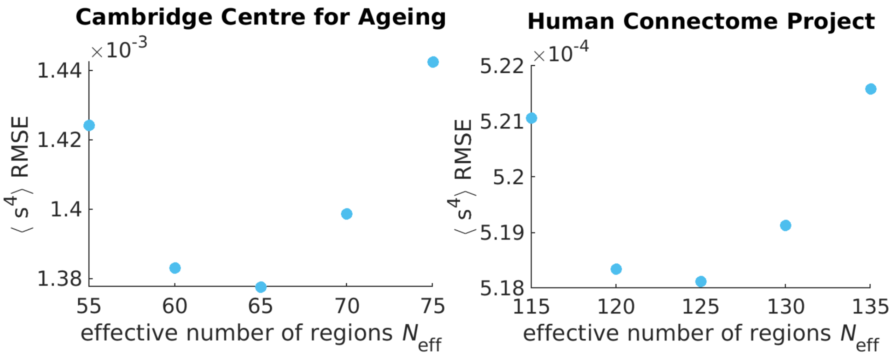

Figure S2. Identifying the effective number of regions N_{eff} for brain scans processed at the voxel-level. Each data point corresponds to the sum over all individuals' RMSEs in the respective data set.

bioRxiv preprint doi: https://doi.org/10.1101/2023.11.17.567376; this version posted May 2, 2024. The copyright holder for this preprint (which was not certified by peer review) is the author/funder, who has granted bioRxiv a license to display the preprint in perpetuity. It is made available under aCC-BY 4.0 International license.

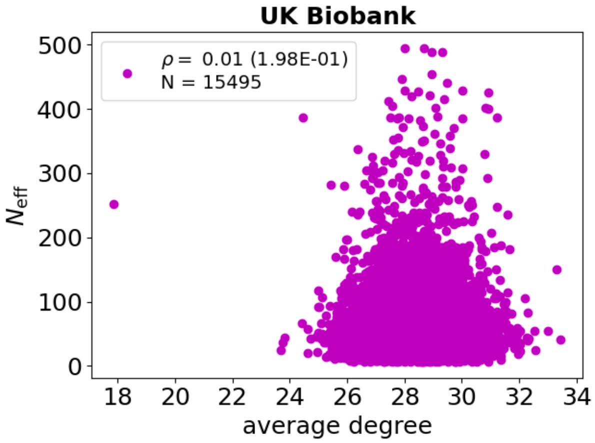

Figure S3. Individually fitted N_{eff} values from Figure S1 are not related to the average number of white matter tracts per brain region (average degree) as determined by diffusion MRI. The Q-Ball tractography method is used to analyze diffusion MRI scans (Methods). Data points correspond to individuals. The variable ρ corresponds to the Spearman correlation coefficient between average degree and N_{eff} calculated over all N individuals, with the p-value in parenthesis.

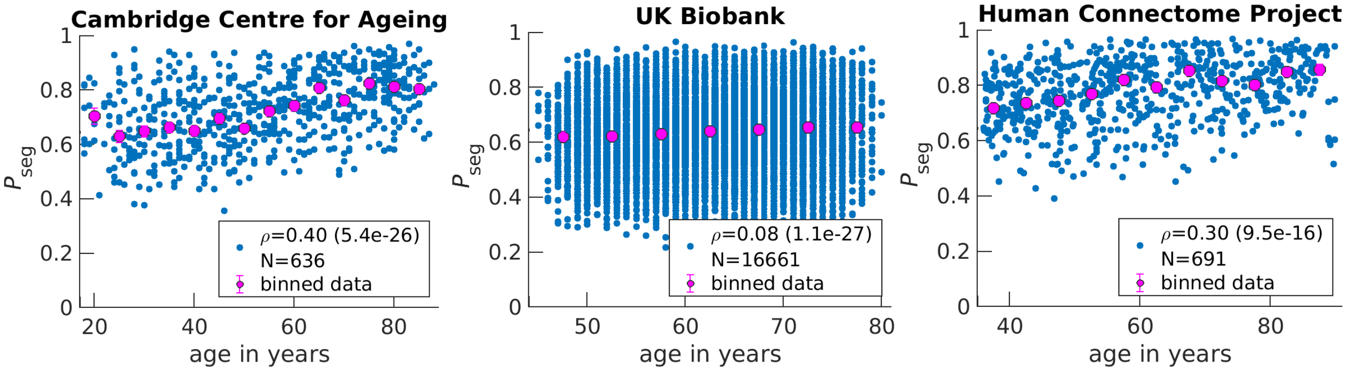

Figure S4. P_{seg} rises on average in aging brains but varies greatly among individuals with the same age. Blue data points correspond to individuals. The variable ρ corresponds to the Spearman correlation coefficient between age and P_{seg} calculated over all N individuals, with the p-value in parenthesis. Magenta points are the exact same data points presented in Figure 3 for the corresponding data set. Note that the corresponding error bars are not visible in these plots.

bioRxiv preprint doi: https://doi.org/10.1101/2023.11.17.567376; this version posted May 2, 2024. The copyright holder for this preprint (which was not certified by peer review) is the author/funder, who has granted bioRxiv a license to display the preprint in perpetuity. It is made available under aCC-BY 4.0 International license.

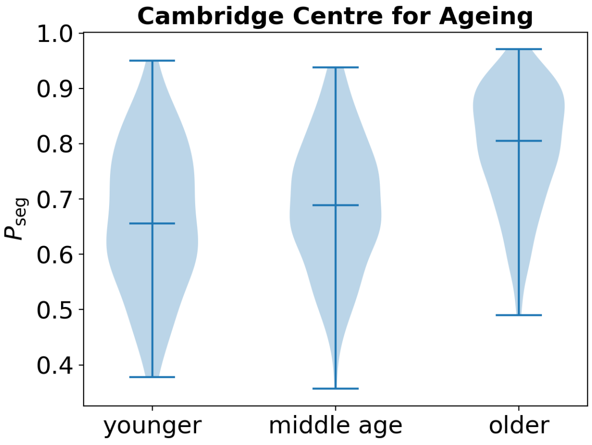

Figure S5. P_{seg} rises in aging brains across three Cambridge Centre for Ageing and Neuroscience age groups. Violin plots are presented, where middle horizontal lines correspond to medians while lower and upper lines correspond to minimum and maximum values, respectively. Younger individuals are those less than 35 years old (N=117); middle age, 40-60y (N=187); older, above 65y (N=209).

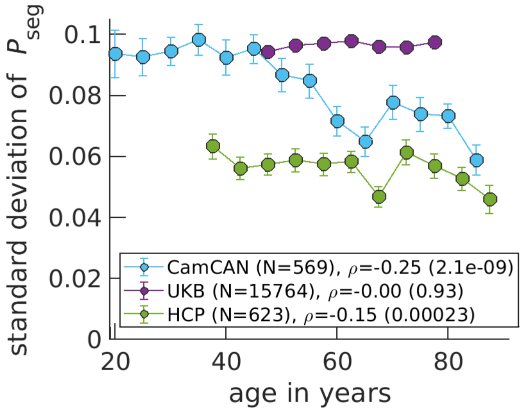

Figure S6. Standard deviations of P_{seg} per individual decreases as a function of age for CamCAN and HCP data sets. Data points correspond to medians, while error bars correspond to standard errors for bins of 5 years. The variable ρ corresponds to the Spearman correlation coefficient between age and P_{seg} calculated over all N individuals, with the p-value in parenthesis. Here, fMRI time-series data for an individual are equally split into 5 chunks and P_{seg} is calculated for each chunk before taking its standard deviation. In the main text, fMRI data are not split up and the entire time-series is considered in calculating P_{seg}.

bioRxiv preprint doi: https://doi.org/10.1101/2023.11.17.567376; this version posted May 2, 2024. The copyright holder for this preprint (which was not certified by peer review) is the author/funder, who has granted bioRxiv a license to display the preprint in perpetuity. It is made available under aCC-BY 4.0 International license.

Table S2. Linear regression results for P_{seg} as a function of age

CamCAN	coefficient	t statistic	Prob> $\|t\|$
intercept	0.575	40.0	8.66E-176
age	0.0028	11.1	3.03E-26

UK Biobank	coefficient	t statistic	Prob> $\|t\|$
intercept	0.556	69.1	<1E-300
age	0.0013	9.92	4.06E-23

HCP	coefficient	t statistic	Prob> $\|t\|$
intercept	0.650	38.1	1.08E-171
age	0.0021	7.58	1.14E-13

Table S3. Multiple linear regression results for P_{seg} as a function of age and sex across the data sets.

CamCAN	coefficient	t statistic	Prob> $\|t\|$
intercept	0.583	39.0	1.22E-170
sex(T.male)	-0.0163	-1.75	8.03E-02
age	0.0028	11.1	1.58E-26

UK Biobank	coefficient	t statistic	Prob> $\|t\|$
intercept	0.561	70.8	<1E-300
sex(T.male)	-0.0445	-23.7	6.17E-122
age	0.0015	12.1	8.79E-34

HCP	coefficient	t statistic	Prob> $\|t\|$
intercept	0.668	39.6	1.92E-179
sex(T.male)	-0.0500	-6.17	1.20E-09
age	0.0022	8.03	4.21E-15

Table S4. Multiple linear regression results for P_{seg} as a function of age, sex and handedness for the Human Connectome Project.

HCP	coefficient	t statistic	Prob> $\|t\|$
intercept	0.677	32.8	5.75E-143
sex(T.male)	-0.0504	-6.20	9.72E-10
handedness(T.right)	-0.0095	-0.76	4.48E-01
age	0.0022	8.00	5.59E-15

bioRxiv preprint doi: https://doi.org/10.1101/2023.11.17.567376; this version posted May 2, 2024. The copyright holder for this preprint (which was not certified by peer review) is the author/funder, who has granted bioRxiv a license to display the preprint in perpetuity. It is made available under aCC-BY 4.0 International license.

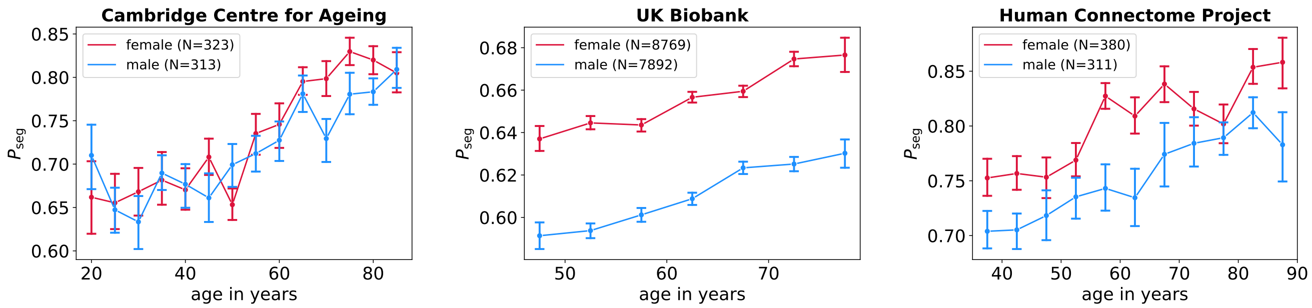

Figure S7. P_{seg} rises in aging brains across three data sets regardless of sex. Data points correspond to medians, while error bars correspond to standard errors for bins of 5 years. For UKB and HCP, we find that females' brains have higher shifted P_{seg} values across age.

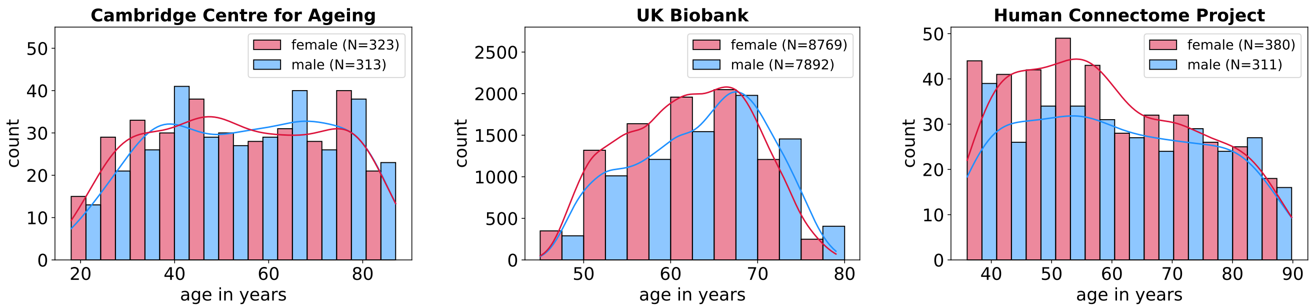

Figure S8. Sex is fairly well-represented across age across the three data sets. Thus, observed P_{seg} aging trends cannot be attributed to the increasing over-representation of one sex.

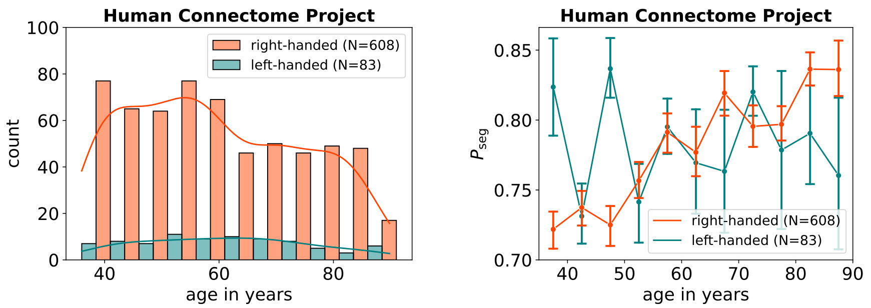

Figure S9. P_{seg} roughly rises in aging brains regardless of handedness. Data points correspond to medians, while error bars correspond to standard errors for bins of 5 years. Large error bars are seen for left-handed individuals because of small sample sizes (left plot).

bioRxiv preprint doi: https://doi.org/10.1101/2023.11.17.567376; this version posted May 2, 2024. The copyright holder for this preprint (which was not certified by peer review) is the author/funder, who has granted bioRxiv a license to display the preprint in perpetuity. It is made available under aCC-BY 4.0 International license.

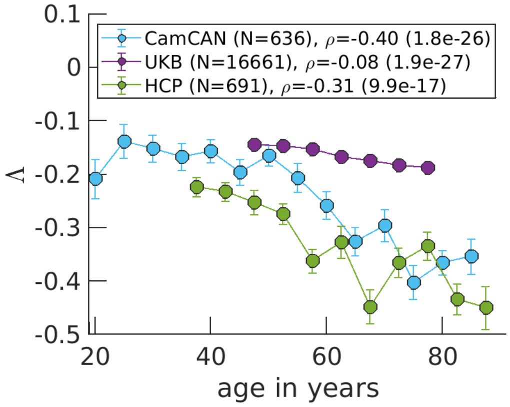

Figure S10. The rescaled connection strength parameter Λ moves further away from the critical point ($\Lambda = 0$) as age increases. Trends are similar in form to Figure 3 because P_{seg} is a function of Λ (Equation 2). Data points correspond to medians, while error bars correspond to standard errors for bins of 5 years. The variable ρ corresponds to the Spearman correlation coefficient between age and Λ calculated over all N individuals, with the p-value in parenthesis.

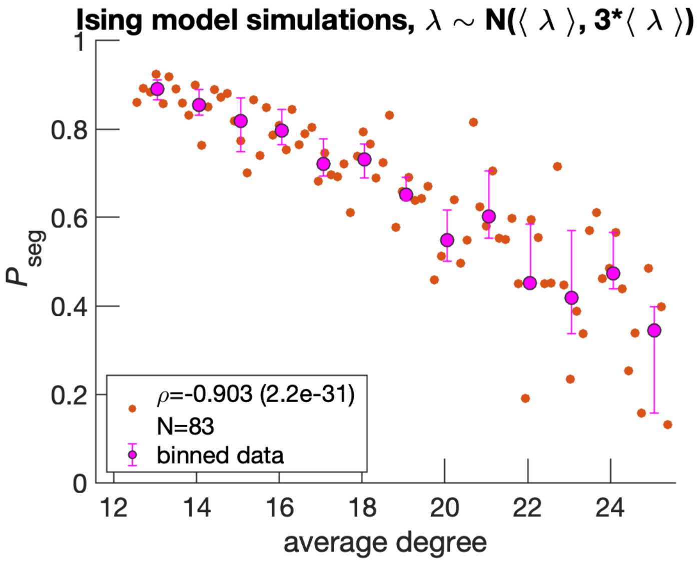

Figure S11. Greater variance in simulations is seen when edges' connection strengths λ are drawn from a normal distribution with mean $\langle\lambda\rangle$ and standard deviation $3 * \langle\lambda\rangle$. At each consecutive step, $\langle\lambda\rangle$ is attenuated such that 5 edges are effectively removed per step ($\langle\lambda'\rangle = \langle\lambda\rangle p_{\text{edge}}$) from the same starting dMRI structure as in Figure 4 (UK Biobank subject ID: 6025360). Data points correspond to medians, while error bars correspond to standard errors for bins of 5 years. Orange data points on the right plot correspond to individual Ising systems, where N reflects the total number. The variable ρ corresponds to the Spearman correlation coefficient calculated over all orange data points between average degree and P_{seg}, with the p-value in parenthesis. Magenta data points correspond to medians, while error bars correspond to upper and lower quartiles for bin sizes of one degree.

bioRxiv preprint doi: https://doi.org/10.1101/2023.11.17.567376; this version posted May 2, 2024. The copyright holder for this preprint (which was not certified by peer review) is the author/funder, who has granted bioRxiv a license to display the preprint in perpetuity. It is made available under aCC-BY 4.0 International license.

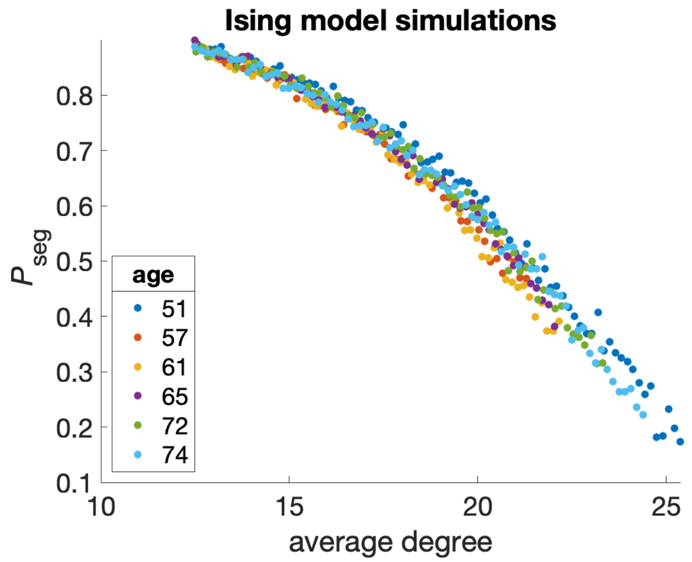

Figure S12. Similar results for Ising simulations are seen as in Figure 4 for different UK Biobank individuals with different ages. Edges are randomly removed as in Figure 4. Starting diffusion MRI structures are used from following subject IDs: 6025360 (51y), 4712851 (57y), 3081886 (61y), 1471888 (65y), 4380337 (72y), and 1003054 (74y).

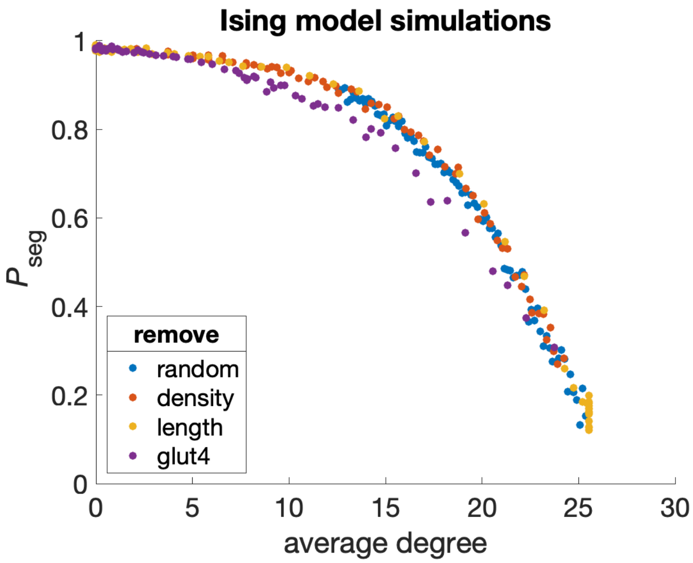

Figure S13. Edge removal mechanisms only matter in so much as they attenuate average degree for Ising simulations. In addition to randomly removing edges as shown in Figure 4, we computationally remove edges based on targeted attack of tract density, tract length, and a node's GLUT4 receptor density. Edges are removed in sequential order, such that those with the largest value are removed first. For all properties except for random, we remove edges until none are present for the same starting dMRI structure as in Figure 4 (UK Biobank subject ID: 6025360).

bioRxiv preprint doi: https://doi.org/10.1101/2023.11.17.567376; this version posted May 2, 2024. The copyright holder for this preprint (which was not certified by peer review) is the author/funder, who has granted bioRxiv a license to display the preprint in perpetuity. It is made available under aCC-BY 4.0 International license.

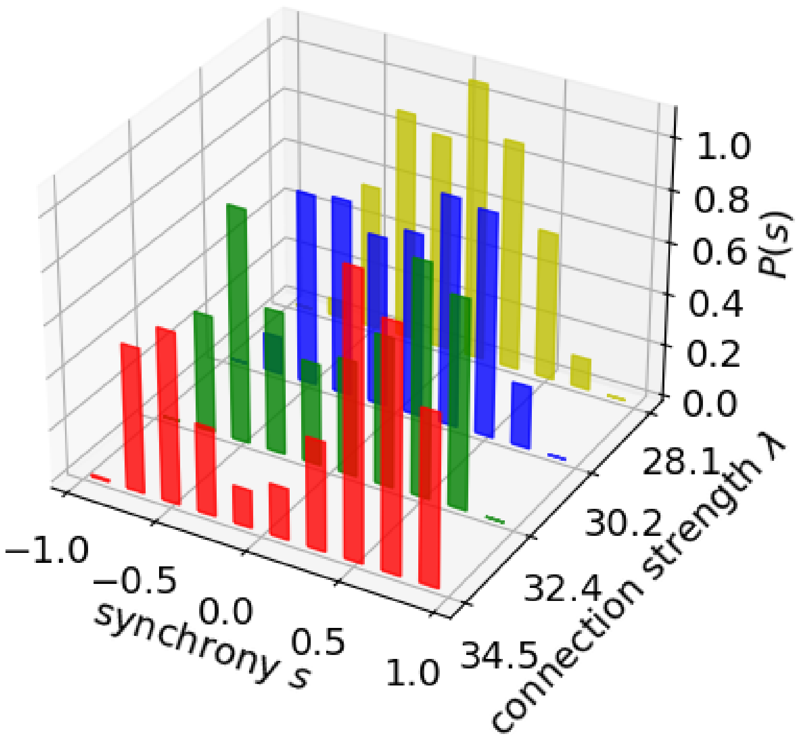

Figure S14. Synchrony distributions transform from bimodal to unimodal as edges are randomly removed from UK Biobank subject ID: 6025360. The parameter λ relates to edge removal because $\lambda = \lambda_0 * p_{\text{edge}}$, where λ_0 is a constant throughout the edge removal process and p_{edge} is the probability that two nodes share an edge (Methods).

bioRxiv preprint doi: https://doi.org/10.1101/2023.11.17.567376; this version posted May 2, 2024. The copyright holder for this preprint (which was not certified by peer review) is the author/funder, who has granted bioRxiv a license to display the preprint in perpetuity. It is made available under a CC-BY 4.0 International license.

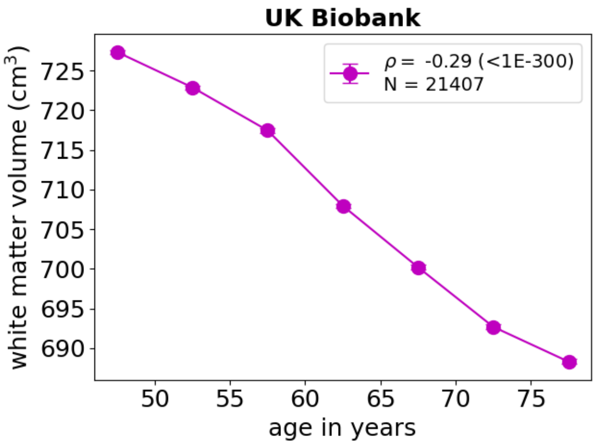

Figure S15. White matter volume decreases with age. White matter volume is measured by structural MRI provided by the UK Biobank. Data points correspond to medians, while error bars correspond to standard errors for bins of 5 years. The variable ρ corresponds to the Spearman correlation coefficient between age and white matter volume calculated over all N individuals, with the p-value in parenthesis. Error bars are plotted but are not visible because of their minuscule size. N is larger than that of Figure 3 because all individuals with structural MRI scans are considered.

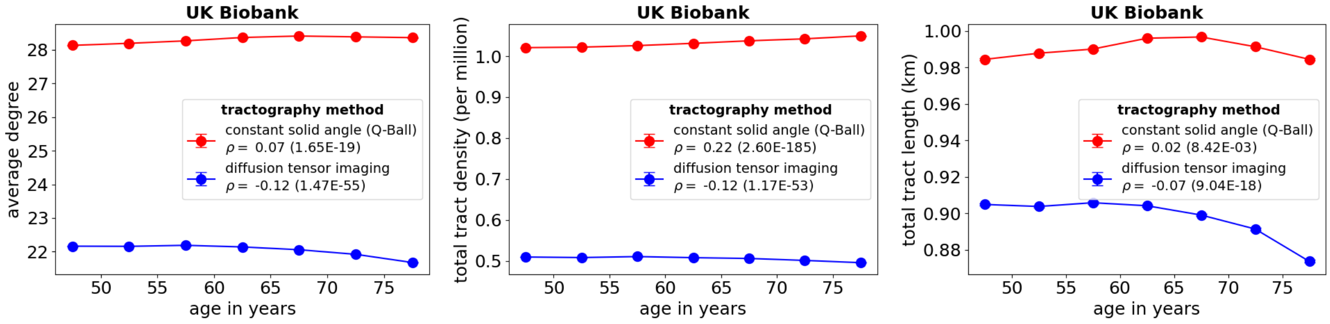

Figure S16. White matter tract properties do not degrade as a function of age when using the Q-Ball method for tractography. However, they do degrade with age when using the less accurate diffusion tensor imaging method. Data points correspond to medians, while error bars correspond to standard errors for bins of 5 years. The variable ρ corresponds to the Spearman correlation coefficient between age and the corresponding property calculated over all available individuals (N=16,649), with the p-value in parenthesis. Error bars are plotted but are not visible because of their minuscule size.

bioRxiv preprint doi: https://doi.org/10.1101/2023.11.17.567376; this version posted May 2, 2024. The copyright holder for this preprint (which was not certified by peer review) is the author/funder, who has granted bioRxiv a license to display the preprint in perpetuity. It is made available under a CC-BY 4.0 International license.

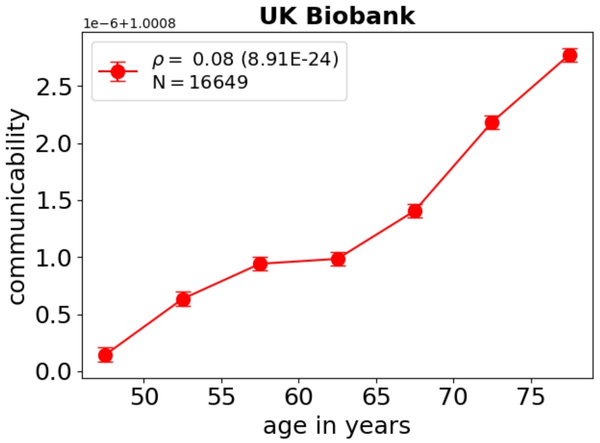

Figure S17. Mean communicability across all brain region pairs does not decrease with age. Communicability is calculated based on tract density as measured by the Q-Ball method for tractography (Methods). Data points correspond to medians, while error bars correspond to standard errors for bins of 5 years. The variable ρ corresponds to the Spearman correlation coefficient between age and white matter volume calculated over all N individuals, with the p-value in parenthesis. Note that the y-axis should be scaled by 10^{-6} and shifted by 1.0008.

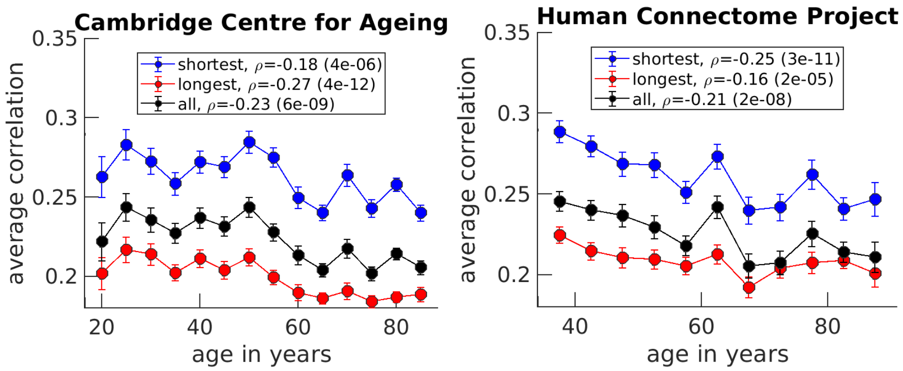

Figure S18. For the Cambridge Centre for Ageing and Neuroscience data set, the shortest edges (lower quartile) have average Pearson correlations or average functional connectivities which correlate less than those of the longest edges (upper quartile). For the Human Connectome Project, the opposite is the case. Edge distances are measured by center of mass coordinates of the brain regions based on the Seitzman atlas. Shortest and longest edges correspond to the lower and upper quartile (25%), respectively. Only positive correlations are considered and diagonal elements are ignored. Data points correspond to medians, while error bars correspond to standard errors for bins of 5 years. The variable ρ corresponds to the Spearman correlation coefficient between age and average correlation calculated over all available individuals ($N_{\mathrm{CamCAN}} = 640$ and $N_{\mathrm{HCP}} = 700$), with the p-value in parenthesis.

bioRxiv preprint doi: https://doi.org/10.1101/2023.11.17.567376; this version posted May 2, 2024. The copyright holder for this preprint (which was not certified by peer review) is the author/funder, who has granted bioRxiv a license to display the preprint in perpetuity. It is made available under aCC-BY 4.0 International license.

Table S5. Functional MRI acquisition parameters of the data sets.

Data set	field strength	repetition time	echo time	flip angle	voxel size	total time points
CamCAN	3T	1970 ms	30 ms	78°	3x3x4.44 mm^3	241
UK Biobank	3T	735 ms	39 ms	52°	2.4x2.4x2.4 mm^3	490
HCP	3T	800 ms	37 ms	52°	2x2x2 mm^3	1912

Table S6. Demographic information of the data sets for those individuals in Figure 3.

Data set	age range	$\langle$age$\rangle$±std(age)	sex
CamCAN	18-87	54.2±18.6	323F/313M
UK Biobank	45-79	54.8±7.4	8769F/7892M
HCP	36-90	59.6±14.9	380F/311M

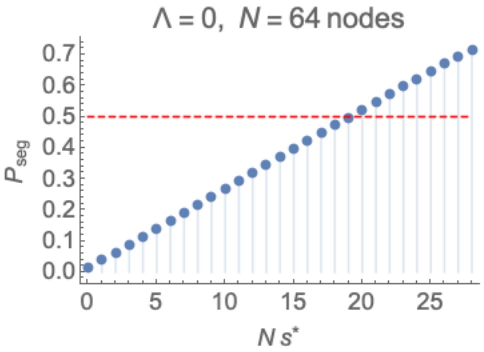

Figure S19. The synchrony threshold s^* is chosen such that it delineates between integrated and segregated states when $P_{\text{seg}} = P_{\text{int}} = 1/2$ (red line) at the critical point ($\Lambda = 0$). This particular figure is created for 64 nodes; it must be set to the corresponding data set's N_{eff} to determine the appropriate synchrony threshold.

bioRxiv preprint doi: https://doi.org/10.1101/2023.11.17.567376; this version posted May 2, 2024. The copyright holder for this preprint (which was not certified by peer review) is the author/funder, who has granted bioRxiv a license to display the preprint in perpetuity. It is made available under a CC-BY 4.0 International license.

TECHNICAL TERMS

Integration a network state composed of global signaling.

Segregation a network state limited to local signaling.

State a particular combination of physical properties. Here, we assume that brain networks can only occupy either the integrated or segregated state.

Ising model a classic model in physics that was first applied to ferromagnetism. It includes pairwise interactions between binary spin states.

Phase interchangeable with the word ‘state’ for the purposes of this text.

Critical Point the point where two phases coexist. In this text, it is where the synchrony distribution dramatically changes from bimodal (primarily integrated) to unimodal (primarily segregated).

Maximum Entropy fit a fitting strategy that satisfies user-defined constraints in the most agnostic way.

White Matter bundles of axons connecting brain regions.
