# Supplementary material for: Brain signaling becomes less integrated and more segregated with age
Source: bioRxiv. 2023 Nov 17:2023.11.17.567376. Preprint. [Version 1] doi: 10.1101/2023.11.17.567376 (PMC10680817; doi:10.1101/2023.11.17.567376)
Supplement: Supplementary file 1 [file NIHPP2023.11.17.567376v1-supplement-1.pdf]

bioRxiv preprint doi: https://doi.org/10.1101/2023.11.17.567376; this version posted November 17, 2023. The copyright holder for this preprint (which was not certified by peer review) is the author/funder, who has granted bioRxiv a license to display the preprint in perpetuity. It is made available under aCC-BY 4.0 International license.

# Supporting Information

## Deriving the Ising model with $N_{\text{eff}}$

Many have derived the probability distribution of the mean field Ising model, otherwise known as the fully connected or Curie-Weiss Ising model [19, 60, 61]. Here, we demonstrate how to introduce $N_{\text{eff}}$ in a Maximum Entropy framework. The tricky part is that $N_{\text{eff}}$ defines the state space over which the probability distribution is summed.

Adding a global pairwise correlation constraint, we obtain the following Lagrangian function over the net displacement of spin states $n = \sum_i^{N_{\text{eff}}} \sigma_i$, where $\sigma_i$ can take a value of 1 or $-1$.

$$\mathcal{L} = \sum_n^{N_{\text{eff}}} P(n) \ln \frac{P(n)}{q(n)} + \alpha \left( \sum_n^{N_{\text{eff}}} P(n) - 1 \right) + \lambda \left( \frac{1}{(N_{\text{eff}})^2} \sum_n^{N_{\text{eff}}} n^2 P(n) - \langle s^2 \rangle_{\text{exp}} \right) \tag{S1}$$

$P$ is the probability distribution. $q$ corresponds to the prior and is set to the binomial distribution $\binom{N}{(N+n)/2} 2^{-N}$, where the binomial coefficient captures the number of ways individual spins can organize for a given $n$. $\alpha$ and $\lambda$ correspond to the Lagrange multipliers that enforce the constraints that the probability

bioRxiv preprint doi: https://doi.org/10.1101/2023.11.17.567376; this version posted November 17, 2023. The copyright holder for this preprint (which was not certified by peer review) is the author/funder, who has granted bioRxiv a license to display the preprint in perpetuity. It is made available under aCC-BY 4.0 International license.

distribution is normalized and the mean pairwise correlation equals $\langle s^2 \rangle$, respectively. The variable $s$ corresponds to the synchrony[5] and is limited to vary from $-1$ to 1 because $n = N_{\text{eff}} s$. This is the reason $N$ does not have to be the same for $\langle s^2 \rangle_{\text{exp}}$ and $\langle s^2 \rangle_{\text{model}}$; $\langle s^2 \rangle$ is always bounded between $-1$ to 1.

Maximizing the Lagrangian function (Equation S1) with respect to $P$, we obtain the following distribution:

$$P(n) = Z^{-1} \binom{N_{\text{eff}}}{(N_{\text{eff}} + n)/2} e^{\lambda n^2 / N_{\text{eff}}^2} \tag{S2}$$

$$P(s) = Z^{-1} \binom{N_{\text{eff}}}{N_{\text{eff}}(1+s)/2} e^{\lambda s^2} \tag{S3}$$

$Z$ corresponds to the partition function and ensures that $P$ is normalized. Note that $Z$ subsumes the $\alpha$ Lagrange multiplier.

## Ising model phase transitions

The Landau model is a general formulation to study phase transitions [62, 20]. It takes the following form,

$$F(T) = A(T - T_c)\eta^2 + B\eta^4 \tag{S4}$$

$\eta$ corresponds to the order parameter. $F$ is the free energy and can be expressed as the probability for being in microstate $i$ by the following relationship $F_i = k_b T \ln P_i$. $T$ is the temperature and $T_c$ corresponds to the critical temperature at which a second-order phase transition occurs. $A$ and $B$ are constants.

Here, we will express the Ising model (Equation S3) in terms of the Landau formalism (Equation S4) by approximating the binomial coefficient as an exponential to $\mathcal{O}(s^4)$. For brevity, we will write $N$ to represent $N_{\text{eff}}$. First, we use Stirling's approximation to expand the binomial coefficient.

$$\binom{N}{N(1+s)/2} = N! \left[ (N(1+s)/2)! \, (N(1-s)/2)! \right]^{-1} \tag{S5}$$

$$\approx N^N \left[ \left( \frac{N(1+s)}{2} \right)^{\frac{N(1+s)}{2}} \left( \frac{N(1-s)}{2} \right)^{\frac{N(1-s)}{2}} \right]^{-1} \tag{S6}$$

$$= N^N \left[ \left( \frac{N}{2} \right)^{\frac{N(1+s)}{2}} (1+s)^{\frac{N(1+s)}{2}} \left( \frac{N}{2} \right)^{\frac{N(1-s)}{2}} (1-s)^{\frac{N(1-s)}{2}} \right]^{-1} \tag{S7}$$

$$= N^N \left[ \left( \frac{N}{2} \right)^N ([1+s][1-s])^{N/2} \left( \frac{1+s}{1-s} \right)^{Ns/2} \right]^{-1} \tag{S8}$$

$$= 2^N \left[ \frac{1}{1-s^2} \left( \frac{1-s}{1+s} \right)^s \right]^{N/2} \tag{S9}$$

To make further headway, we assume $s \approx 0$ and expand Equation S9 to order $s^4$.

$$\binom{N}{N(1+s)/2} \approx 2^N \left[ 1 - s^2 - \frac{1}{3} s^4 \right]^{N/2} \tag{S10}$$

Next, we assume $N$ is large and express the term under the brackets as an exponential.

[5] In ferromagnetic applications, $s$ is commonly referred to as the magnetization.

bioRxiv preprint doi: https://doi.org/10.1101/2023.11.17.567376; this version posted November 17, 2023. The copyright holder for this preprint (which was not certified by peer review) is the author/funder, who has granted bioRxiv a license to display the preprint in perpetuity. It is made available under aCC-BY 4.0 International license.

$$\binom{N}{N(1+s)/2} \approx 2^N \exp\left[-\frac{N}{2}s^2 - \frac{N}{12}s^4\right] \tag{S11}$$

We can insert our approximate expression for the binomial coefficient back into $P(s)$ (Equation S3) and obtain,

$$P(s) \approx Z^{-1}2^N \exp\left[\left(\lambda - \frac{N}{2}\right)s^2 - \frac{N}{12}s^4\right] \tag{S12}$$

Note that Equation S12 (after transforming into free energy space) maps onto Landau theory (Equation S4). $s$ corresponds to the order parameter and $\lambda_c = N/2$. At $\lambda = \lambda_c$, $P(s)$ switches from unimodal to bimodal, corresponding to a second order phase transition. We report a rescaled version of $\lambda$ called $\Lambda$ in Figure S4 and in other places in the Supporting Information to easily gauge how far an individual's connection strength is from the critical point.

$$\Lambda = \frac{\lambda - \lambda_{\text{critical}}}{\lambda_{\text{critical}}} \tag{S13}$$

## Alternative $N_{\text{eff}}$ fitting approach

Rather than choose one $N_{\text{eff}}$ for all individuals in the data set as done in the main text, we could fit $N_{\text{eff}}$ for each individual. Figure S1 demonstrates that such a procedure results in $N_{\text{eff}}$ values that are highly linearly correlated with $\lambda$. In other words, more precise $N_{\text{eff}}$ fits do not provide any more insight than maximum entropy fits of $\lambda$ for all individuals in a data set under one optimal $N_{\text{eff}}$.

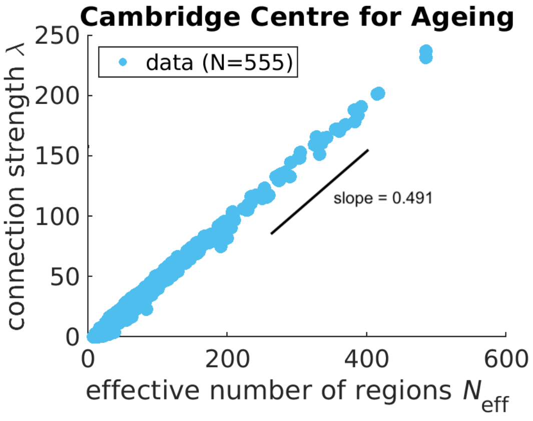


Figure S1: Treating $N_{\text{eff}}$ as a parameter and fitting it per individual yields a strong correlation with $\lambda$. Each point reflects an individual brain scan and N reflects the total number analyzed. The value of the slope corresponds to that of the best-fit line for the data.

The $N_{\text{eff}}$-$\lambda$ relationship can be reasoned from the analytical expression for $P(s)$ (Equation S12). When $\Lambda < 0$, which many individuals satisfy (Figure S4), $P(s)$ is well-approximated as a Gaussian.

$$P(s) \propto \exp\left[\left(\lambda - \frac{N_{\text{eff}}}{2}\right)s^2\right] \tag{S14}$$

Thus, the analytical form for $\langle s^2\rangle$ is:

$$\langle s^2\rangle = -\frac{1}{2\lambda - N_{\text{eff}}} \tag{S15}$$

Since $\lambda$ is fit in the Maximum Entropy framework to exactly match $\langle s^2\rangle$, Equation S15 indicates that a larger $N_{\text{eff}}$ requires a larger $\lambda$ for a fixed $\langle s^2\rangle$. Indeed, we find in Figure S1 that the best fit line of the $N_{\text{eff}}$-$\lambda$ relationship has an approximate slope of 0.5, in agreement with Equation S15.

bioRxiv preprint doi: https://doi.org/10.1101/2023.11.17.567376; this version posted November 17, 2023. The copyright holder for this preprint (which was not certified by peer review) is the author/funder, who has granted bioRxiv a license to display the preprint in perpetuity. It is made available under aCC-BY 4.0 International license.

# Miscellaneous

Additional supplementary tables and figures that are directly referenced in the main text. For Figures presenting data set results, we adopt the following symbol codes unless otherwise noted. Data points correspond to individuals, where N reflects the total number. The variable $\rho$ corresponds to the Spearman correlation coefficient between age and the property of interest (i.e. $P_{\text{seg}}$, white matter volume) calculated over all individuals, with the p-value in parenthesis. Error bars correspond to standard errors for equally spaced bins.

Table S1: Data set values for $P_{\text{seg}}$ calculation.

| Data set | effective number of regions $N_{\text{eff}}$ | synchrony threshold $s^*$ |
|---|---|---|
| Cambridge Centre for Ageing | 40 | 0.334 |
| UK Biobank | 30 | 0.357 |
| Human Connectome Project | 40 | 0.334 |

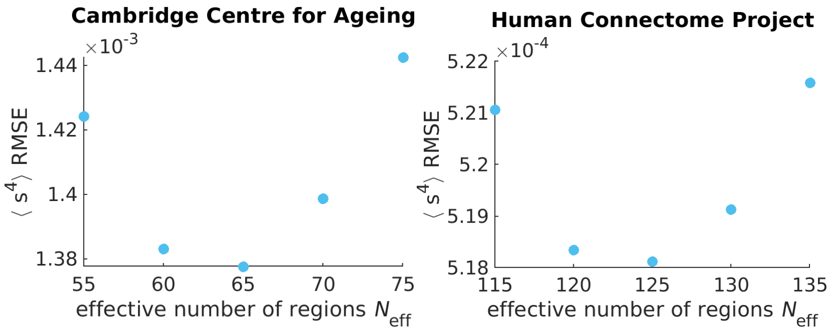


Figure S2: Identifying the effective number of regions $N_{\text{eff}}$ for brain scans preprocessed at the voxel-level. Each data point corresponds to the sum over all individuals' RMSEs in the respective data set.

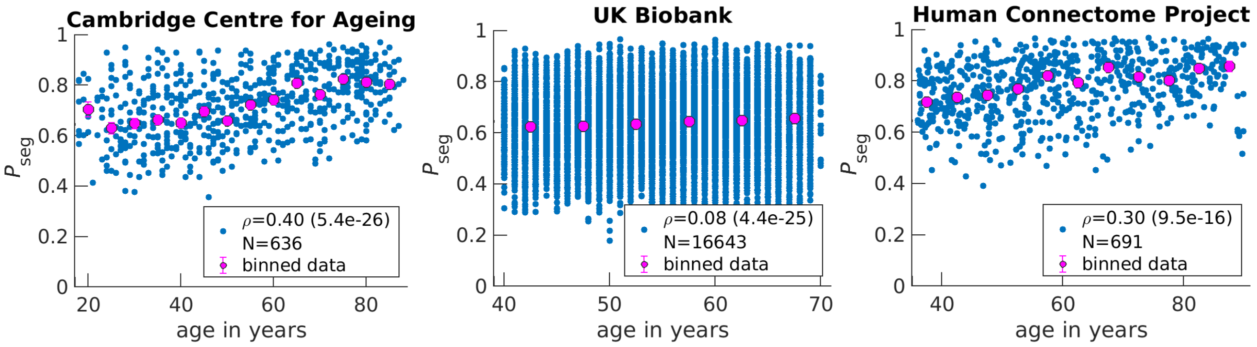


Figure S3: $P_{\text{seg}}$ rises in aging brains across three data sets. Blue data points correspond to individuals, where N reflects the total number. The variable $\rho$ corresponds to the Spearman correlation coefficient between age and $P_{\text{seg}}$ calculated over all individuals, with the p-value in parenthesis. Magenta data points correspond to medians, while error bars correspond to standard errors for bins of 5 years.

bioRxiv preprint doi: https://doi.org/10.1101/2023.11.17.567376; this version posted November 17, 2023. The copyright holder for this preprint (which was not certified by peer review) is the author/funder, who has granted bioRxiv a license to display the preprint in perpetuity. It is made available under aCC-BY 4.0 International license.

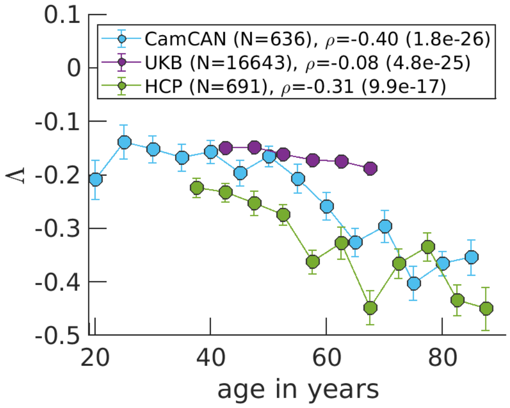


Figure S4: The rescaled connection strength parameter $\Lambda$ moves further away from the critical point ($\Lambda = 0$) as age increases. Trends are similar in form to Figure 3 because $P_{\text{seg}}$ is a function of $\Lambda$.

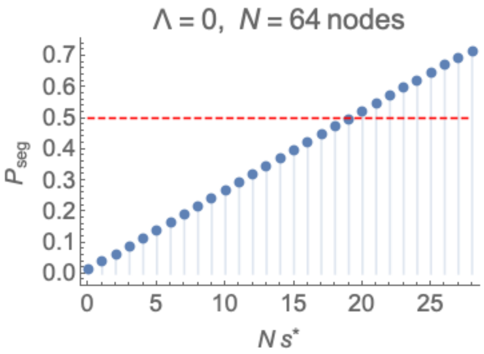


Figure S5: The synchrony threshold $s^*$ is chosen such that it delineates between integrated and segregated states when $P_{\text{seg}} = P_{\text{int}} = 1/2$ (red line) at the critical point ($\Lambda = 0$). This particular figure is created for 64 nodes; it must be set to the corresponding data set's $N_{\text{eff}}$ to determine the appropriate synchrony threshold.

bioRxiv preprint doi: https://doi.org/10.1101/2023.11.17.567376; this version posted November 17, 2023. The copyright holder for this preprint (which was not certified by peer review) is the author/funder, who has granted bioRxiv a license to display the preprint in perpetuity. It is made available under aCC-BY 4.0 International license.

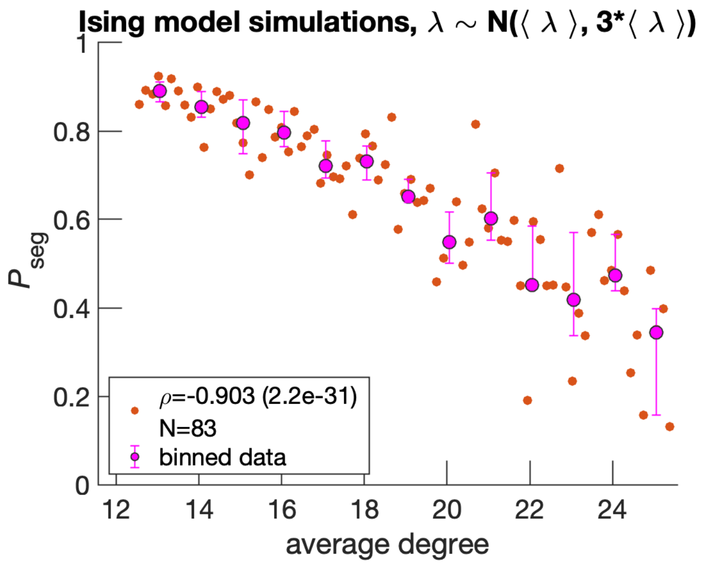


Figure S6: Greater variance in simulations is seen when edges' connection strengths $\lambda$ are drawn from a normal distribution with mean $\langle\lambda\rangle$ and standard deviation $3 * \langle\lambda\rangle$. At each consecutive step, $\langle\lambda\rangle$ is attenuated such that 5 edges are removed per step from the same starting dMRI structure as in Figure 4 ($\langle\lambda'\rangle = \langle\lambda\rangle p_{\text{edge}}$).

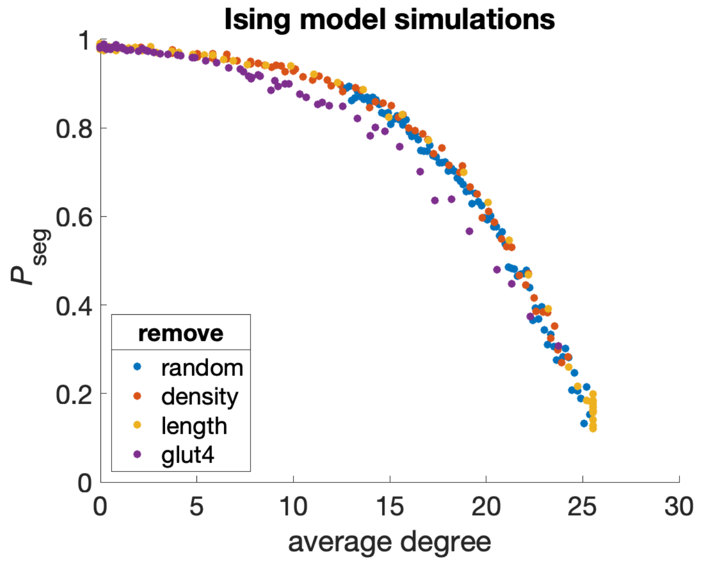


Figure S7: Edge removal mechanisms only matter in so much as they attenuate average degree. In addition to randomly removing edges as shown in Figure 4, we remove edges based on targeted attack of tract density, tract length, and a node's GLUT4 receptor density. Edges are removed in sequential order, such that those with the largest value are removed first. For all properties except for random, we remove edges until none are present.

bioRxiv preprint doi: https://doi.org/10.1101/2023.11.17.567376; this version posted November 17, 2023. The copyright holder for this preprint (which was not certified by peer review) is the author/funder, who has granted bioRxiv a license to display the preprint in perpetuity. It is made available under aCC-BY 4.0 International license.

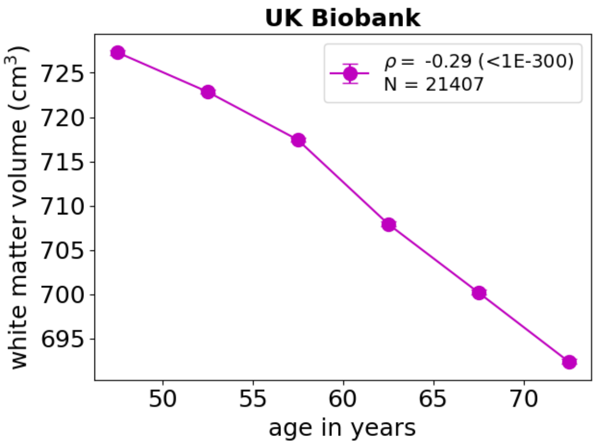


Figure S8: White matter volume decreases with age. White matter volume is measured by structural MRI provided by the UK Biobank. Error bars that represent standard errors are plotted but are not visible because of their minuscule size.

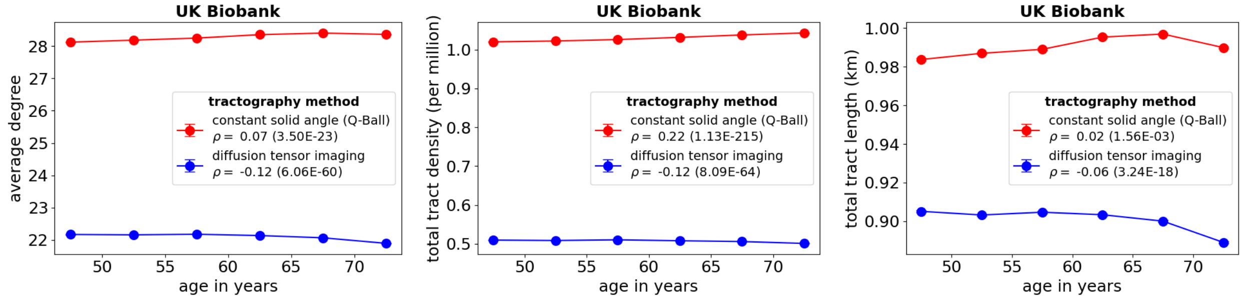


Figure S9: White matter tract properties do not degrade as a function of age when using the Q-Ball method for tractography. However, they do degrade with age when using the less accurate diffusion tensor imaging method. Error bars that represent standard errors are plotted but are not visible because of their minuscule size.

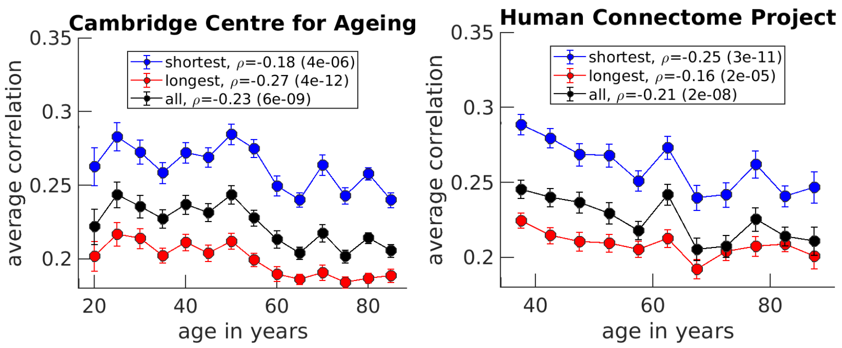


Figure S10: For the Cambridge Centre for Ageing data set, the shortest edges (lower quartile) have average Pearson correlations which correlate weaker than those of the longest edges (upper quartile). For the Human Connectome project, the opposite is the case. Edge distances are measured by center of mass coordinates of the brain regions based on the Seitzman atlas. Shortest and longest edges correspond to the lower and upper quartile (25%), respectively. Only positive correlations are considered and diagonal elements are ignored.

bioRxiv preprint doi: https://doi.org/10.1101/2023.11.17.567376; this version posted November 17, 2023. The copyright holder for this preprint (which was not certified by peer review) is the author/funder, who has granted bioRxiv a license to display the preprint in perpetuity. It is made available under aCC-BY 4.0 International license.

Table S2: Functional MRI acquisition parameters of the data sets.

| Data set | field strength | repetition time | echo time | flip angle | voxel size | total time points |
|---|---|---|---|---|---|---|
| CamCAN | 3T | 1970 ms | 30 ms | 78° | 3x3x4.44 $mm^3$ | 241 |
| UK Biobank | 3T | 735 ms | 39 ms | 52° | 2.4x2.4x2.4 $mm^3$ | 490 |
| HCP | 3T | 800 ms | 37 ms | 52° | 2x2x2 $mm^3$ | 1912 |
